# Diagnosing Gestational Diabetes by a Single-Test Procedure Is a Propitious Step Towards Containing the Epidemic of Diabetes

**DOI:** 10.7759/cureus.19910

**Published:** 2021-11-25

**Authors:** V Seshiah, Vijayam Balaji, Stephen C Bronson, Rajesh Jain, Anjalakshi Chandrasekar

**Affiliations:** 1 Diabetology, The Tamil Nadu Dr. M.G. Ramachandran Medical University, Chennai, IND; 2 Diabetology, Dr. V. Balaji Dr. V. Seshiah Diabetes Care and Research Institute, Chennai, IND; 3 Diabetology, Stanley Medical College and Hospital, Chennai, IND; 4 Public Health, Jain Hospital and Research Center, Kanpur, IND; 5 Obstetrics and Gynecology, Madha Medical College & Research Institute, Chennai, IND

**Keywords:** universal screening, cost-efficiency, cost-effectiveness, diagnosis, screening, gdm, hyperglycemia in pregnancy, single test procedure, gestational diabetes

## Abstract

In recent years, diabetes has evolved into a non-communicable disease pandemic with data showing that one out of ten adults in the world have diabetes. Among various factors that contribute to this rising trend in diabetes, one factor that is of paramount importance is gestational diabetes mellitus (GDM). Maternal hyperglycemia sets off a vicious cycle that affects not only the mother and her child but also the generations to come. There are many criteria that are used for the diagnosis of GDM. Almost all of these criteria require the pregnant woman to be in the fasting state in order to perform an oral glucose tolerance test (OGTT). In many parts of the world, especially in low- and middle-income countries, OGTT is a resource-intensive and technically demanding procedure. More often than not, pregnant women do not attend the antenatal clinic fasting. If they are asked to come fasting again for the OGTT, the drop-out rate is increased. Thus, for practical purposes, a test that is feasible on the ground is essential. In this paper, we emphasize a "single-test" procedure wherein a 75-gram oral glucose load is administered to the pregnant woman irrespective of whether she is in the fasting state or not, and plasma glucose is measured at two hours. A plasma glucose value ≥ 140 mg/dL (7.8 mmol/L) at two hours is considered diagnostic of GDM. The single-test procedure was found to be a sustainable, cost-effective, evidence-based, and affordable test procedure for any society. It serves both as a screening test and a diagnostic test for GDM. Furthermore, we emphasize the need for universal screening of all pregnant women who attend the antenatal clinics to detect dysglycemia, especially in the early weeks of pregnancy when the impact on the growing fetus would be significant.

## Introduction

Diabetes is a global public health problem. Recent data from the International Diabetes Federation (IDF) shows a continued increasing trend in the prevalence of diabetes all over the world, such that one in 10 adults in the world live with diabetes and almost half of them are undiagnosed. Health expenditure due to diabetes has seen a 316% increase in the last 15 years amounting to nearly United States Dollar (USD) 966 billion. It is observed that diabetes is hitting the poorest, hardest - as four out of five people with diabetes live in low- and middle-income countries. Remarkably, diabetes in recent years has become a non-communicable disease pandemic with significant adverse impact [1,2].

Even though several other factors contribute towards the increasing prevalence of diabetes in the world, one area that deserves considerable attention is diabetes related to pregnancy, termed Hyperglycemia in Pregnancy (HIP). HIP includes pure gestational diabetes mellitus (GDM), overt-diabetes detected during pregnancy also known as Diabetes in Pregnancy (DIP), and pregnant women with pre-existing diabetes. HIP has adverse consequences beyond the index pregnancy, as the mothers are vulnerable to developing type 2 diabetes later in life and their offspring are predisposed to develop obesity, insulin resistance (IR), diabetes, hypertension and cardiovascular diseases in adulthood. On the lines of Barker’s hypothesis of "fetal origins of adult disease", maternal hyperglycemia acts as an intrauterine insult to the developing fetus, setting off a vicious cycle of transgenerational transmission of diabetes and related non-communicable diseases [3].

The diagnostic criteria adapted for the diagnosis of GDM vary considerably among nations and also among different regions within a country. This paper examines how a single-test procedure would be sustainable and cost-effective for the diagnosis of GDM at the earliest possible time in pregnancy.

## Technical report

Practical problems in diagnosing GDM

Various diagnostic criteria exist around the world for diagnosing GDM [4]. Of these, the guideline proposed by the International Association of Diabetes and Pregnancy Study Groups (IADPSG) is widely used. However, almost every diagnostic criterion, including that of IADPSG, requires pregnant women to be fasting for the oral glucose tolerance test (OGTT) to be performed [5]. OGTT is a relatively complex, multi-step procedure, and many healthcare systems, especially in lower-income regions of the world, might not be able to carry out an OGTT in pregnant women as part of an antenatal checkup. In such cases, many centers might not screen for hyperglycemia in pregnancy [5]. Hence, diagnostic techniques that do not need a multi-step OGTT to detect GDM are preferable. In addition, most pregnant women do not come fasting, for their routine antenatal visit. When they are asked to come again fasting, the drop-out rate is high because of the cost and time involved [6]. Furthermore, for a pregnant woman, the demand to be fasting while attending the clinic or laboratory for a blood test could be quite uncomfortable because of the time taken to cover long distances to the test centers in many regions, even in developed countries [7]. A blood test in the fasting state during pregnancy, especially in the early months, is often troublesome due to increased nausea. Thus, when there is a necessity to be fasting for GDM testing, the drop-out frequency is high when a pregnant lady who had not been fasting in her first visit is asked to come again for the OGTT [8]. Consequently, considering the above facts, a non-fasting test is more desirable in the pregnant state [5]. Hence a need arose to evaluate a test that could be performed without imposing any restrictions to undertake the test.

The "single-test procedure"

In line with the earlier observations by Pettitt et al. [9] and Franks et al. [10], a diagnostic test procedure for GDM in which the pregnant woman need not necessarily be fasting had been evaluated and reported by Anjalakshi et al. [11]. This is called the "single-test procedure" and is described as follows.

In this technique, the pregnant woman (who is not known to have pre-existing diabetes) who comes to visit the antenatal clinic is administered a 75-g oral glucose load, whether she is in the fasting state or non-fasting state, without regard to the timing of her preceding meal. The 75-g glucose is to be given orally after dissolving in approximately 300 ml of water. The ingestion of the solution is to be completed within five minutes. A plasma-standardized glucometer should be used to check blood glucose two hours after the glucose load. A threshold plasma glucose (PG) value of more than or equal to 140 mg/dL (7.8 mmol/L) is taken as the cut-off for the diagnosis of GDM. If the test is negative in the first trimester, then it is repeated in subsequent trimesters. This test is also known as a "non-fasting 75-g OGTT" and also as the DIPSI procedure after the Diabetes-in-Pregnancy Study Group-India (DIPSI), a body that endorses and advocates the procedure [4].

The reasoning behind the concept of this test is that a woman with normal insulin secretion and/or insulin sensitivity should be able to tolerate a glucose load and maintain normoglycemia, irrespective of the timing of her last meal. This is because of the brisk and sufficient insulin response in such a woman with normal glucose tolerance. On the other hand, in a woman who has GDM, the blood glucose level increases after a meal [12] and when subjected to a glucose challenge, the blood glucose level gets amplified further. This is because of the impaired insulin secretion in a woman with GDM. Furthermore, a woman who exhibits glucose intolerance during pregnancy is also at risk to develop diabetes in the future. 

The test is recommended to be done at the first visit of the pregnant woman to the antenatal clinic irrespective of whether she is fasting or not. This is to avoid the need of asking her to come another day in the fasting state for an OGTT, which is likely to increase the drop-out rate [8]. Furthermore, given the alarming trend in the increasing prevalence of type 2 diabetes in the world, and the need to identify glycemic perturbations in the early weeks of pregnancy, it is recommended to do "universal screening" of all pregnant women for GDM by this convenient single-test procedure, even in the early weeks of pregnancy, rather than performing a risk-factor based screening at around 24-28 weeks using a multi-step OGTT [8]. Diagnosis of GDM with 2-h PG ≥ 140 mg/dl (7.8 mmol/L) and treatment are beneficial with a decrease in the incidence of macrosomia, decreased perinatal complications, and cesarean deliveries [13].

## Discussion

Advantages of the single-test procedure

The benefits of this single-test procedure are as follows: the pregnant woman need not necessarily be fasting for the test; the test procedure is simple and not cumbersome that it does not affect much, the day-to-day routine of the pregnant woman; the test is a screening as well as a diagnostic tool for GDM (thereby enabling easy universal testing of all pregnant women at the earliest visit, which is especially beneficial in regions and ethnicities with a high prevalence of diabetes); beneficial for diagnosing GDM in the present context of COVID-19 pandemic where movement is restricted and man-power is limited. Furthermore, in resource-limited settings as in developing countries, laboratory facilities for testing blood glucose are less likely to be widely available, and therefore, in this procedure, using a portable glucometer standardized to be on par with PG levels is recommended [5]. In addition, it is noteworthy that in randomized controlled trials where the advantages of treating GDM were demonstrated, the identification of those with GDM was done mainly by using PG values after a glucose load. In addition, there is no high-standard observational evidence to say that pregnant women and their offspring derive appreciable benefit from treatment if only the fasting PG level is abnormal [5].

Cost-effectiveness of the single-test procedure in gestational diabetes screening

The single-test procedure for the diagnosis of GDM requires only one blood sample obtained after a 75-g oral glucose load. Even in case if the test is negative in the first trimester and it needs to be done again in subsequent trimesters, the cost of this procedure would be lesser than that of the IADPSG procedure (which is a multiple-step approach), by about 66%. Screening strategy based on the IADPSG criteria may be cost-effective for high resource settings (USD 61,503/Quality Adjusted Life Years) but probably is too costly for most countries [14]. Therefore, for successful implementation of GDM screening with due regard to local priorities, cost-efficiency of the procedure and availability of required resources need to be considered.

Marseille et al. analyzed the cost-effectiveness of GDM screening and prevention of type 2 diabetes, in two countries - India and Israel [15]. They observed that GDM without intervention resulted in higher disability-adjusted life years (DALYs) when compared to GDM with intervention. Comparing the price of illness concerning the years lost to disability in both the groups, it was observed that GDM patients who undertook intervention spent lesser compared to those patients without any intervention. The critical result of this study is price effectiveness. This was calculated by evaluating the incremental cost of preventing complications concerning the number of years of disability saved, and the resultant value is USD 1626. Based on the recommendation of WHO on price effectiveness, it has been established that any program is said to be highly cost-effective if the charge is smaller than the per capita GDP of that country. Accordingly, screening for GDM with a single-test procedure in the antenatal period is a cost-effective strategy for preventing perinatal complications and the long-term post-partum risk of type 2 diabetes in both the mother and child. 

Nielsen et al. demonstrated that minimizing complex, step-wise processes is important in expanding GDM screening everywhere, especially in low- and middle-income countries. Furthermore, they showed that in GDM screening, techniques that are simple, practical, easily applicable in the field, yet rigorous, are essential [16]. Thus, the single-test procedure is a possible, sustainable, cost-effective, evidence-based, and high-impact affordable test procedure for any society. This procedure is recommended by the Health Ministry of India [13] and recognized by international bodies like IDF [4] and the International Federation of Gynecology and Obstetrics (FIGO) [6].

Prevention of diabetes

It is advisable to screen for glucose tolerance in the first trimester itself. This is essential as all the vital organs develop during this period of gestation. Early pregnancy exposure to surplus maternal fuels may impact placental transportation in a time-dependent manner. Metabolic perturbations are underway before the usual time of diagnosis (which is done by screening at 24 to 28 weeks), and hence, earlier screening and intervention are warranted. GDM may manifest itself in all trimesters of pregnancy. Therefore, there is a pressing need for testing glucose tolerance in the early weeks of pregnancy [3].

The peri-conceptional and intra-uterine periods and childhood are crucial time periods to be targeted for lifestyle and behavioral interventions for the prevention of diabetes. Pregnancy offers a vital opportunity to develop, test, and implement clinical strategies for the prevention of diabetes and other non-communicable diseases. Universal screening of all pregnant women, even from the early weeks of pregnancy for GDM, and achieving optimum PG control, while ensuring adequate maternal nutrition, is highly likely to prevent and slow down the vicious cycle of passing on glucose intolerance from generation to generation [3]. Hence, it can be concluded that a highly efficient way to alleviate the epidemic of diabetes is to “Focus on the Fetus for the Future” (fondly called "Seshiah’s dictum" by colleagues after the senior author of this paper).

## Conclusions

GDM is known to be associated with maternal and fetal complications during pregnancy and post-natal periods. A woman with GDM carries an increased risk of developing frank diabetes in the future. Furthermore, due to adverse in-utero metabolic programming, the offspring carries a risk of developing obesity and diabetes in adolescence and adulthood. Therefore, preventing GDM by effective lifestyle interventions starting from early childhood and thereby maintaining a healthy intra-uterine metabolic milieu would go a long way in preventing the transgenerational transmission of diabetes. To achieve this purpose, a simple, practical, and effective screening/diagnostic test for GDM is essential.

In this report, we have described the importance and practicality of the "single-test procedure" that can efficiently be implemented in the process of screening and diagnosing GDM. Furthermore, we have emphasized an early universal GDM screening for all pregnant women who attend the antenatal clinic. Testing for GDM at the very first visit is recommended starting from the early weeks of pregnancy.

We conclude this paper by quoting Norbert Freinkel, a pioneer in GDM: “No single period in human development provides a greater potential than pregnancy for long-range pay-off via a relatively short-range period of enlightened metabolic manipulation.”
